# Gene expression profiling in a mouse model of retinal vein occlusion induced by laser treatment reveals a predominant inflammatory and tissue damage response

**DOI:** 10.1371/journal.pone.0191338

**Published:** 2018-03-12

**Authors:** Gottfried Martin, David Conrad, Bertan Cakir, Günther Schlunck, Hansjürgen T. Agostini

**Affiliations:** Eye Center, Medical Center, Medical Faculty, University of Freiburg, Freiburg, Germany; Children's Hospital Boston, UNITED STATES

## Abstract

**Purpose:**

Retinal vein occlusion (RVO) has been investigated in several laser-induced animal models using pigs, rabbits and rats. However, laser-induced RVO has been rarely reported in mice, despite the impressive number of available mutants, ease of handling and cost effectiveness. The aim of this study was to further assess the feasibility of a RVO mouse model for gene expression analysis and its possible use to investigate effects of hypoxia.

**Methods:**

C57Bl/6J mice were injected with eosin Y for photo-sensitization. Subsequently, large retinal veins were laser-treated in one eye to induce vascular occlusion. Contralateral control eyes received non-occlusive retinal laser treatment sparing large vessels. The animals were followed for up to eight days and assessed by funduscopy, angiography, hypoxyprobe staining, histopathology and gene expression analysis by qPCR and RNA sequencing (RNAseq). Another group of mice was left untreated and studied at a single time point to determine baseline characteristics.

**Results:**

Laser-induced RVO persisted in half of the treated veins for three days, and in a third of the veins for the whole observation period of 8 days. Funduscopy revealed large areas of retinal swelling in all laser-treated eyes, irrespective of vascular targeting or occlusion status. Damage of the outer retina, retinal pigment epithelium (RPE), and even choroid and sclera at the laser site was observed in histological sections. Genes associated with inflammation or cell damage were highly up-regulated in all laser-treated eyes as detected by RNAseq and qPCR. Retinal hypoxia was observed by hypoxyprobe staining in all RVO eyes for up to 5 days with a maximal extension at days 2 and 3, but no significant RVO-dependent changes in gene expression were detected for angiogenesis- or hypoxia-related genes.

**Conclusion:**

The laser-induced RVO mouse model is characterized by a predominant general inflammatory and tissue damage response, which may obscure distinct hypoxia- and angiogenesis-related effects. A non-occlusive laser treatment control is essential to allow for proper data interpretation and should be mandatory in animal studies of laser-induced RVO to dissect laser-induced tissue damage from vascular occlusion effects.

## Introduction

Retinal vein occlusion (RVO) is a common vascular eye disease that causes vision loss due to macular edema, retinal bleeding and ischemia [1,2]. Rare clinical studies examined concentrations of different proteins in vitreous samples of RVO patients [3]. Appropriate animal models to study tissue regeneration following RVO are still being developed.

Earlier reports on RVO animal models provided mixed results. Direct ligation of retinal veins is difficult and only feasible in large animals. In mice, ligation of both, central retinal artery and vein just outside the eye was reported to result in a stroke-like phenotype of whole eye malperfusion [4]. Injection of thrombin (F2a) near a retinal vein in rabbits resulted in a rather brief vascular occlusion [5], and injection of endothelin-1 (ET-1) induced occlusions lasting a few hours at best [6]. The most commonly reported method to occlude retinal veins is the intravenous injection of a photosensitizier such as rose bengal, fluorescein, erythrosin B, or neutral red and subsequent localized activation by an appropriate laser treatment. This strategy appears to work well in pigs [7–11], rabbits [12,13], and rats [14–17].

In studies with murine RVO models [18–26], vascular occlusion was observed by funduscopy as interrupted blood flow at the laser site and as edema detected by fluorescence angiography. Some days after laser treatment, non-perfused retinal areas and vascular leakage were detected. Retinal swelling was detected in OCT images and dilated and tortuous vessels in retinal flatmounts. Structures resembling vascular tufts were reported 2–3 weeks after laser treatment. Laser-induced damage was evaluated in histological sections, but reports characterizing hypoxia were missing.

The aim of our present study was to investigate distinct hypoxia-dependent gene expression changes in the murine laser-induced retinal vein occlusion model. However, employing a non-occlusive control laser treatment, our data revealed a general laser-induced upregulation of inflammatory and wounding-related genes rather than distinct alterations of hypoxia- or angiogenesis-related genes. This would have been missed using untreated control eyes for comparison as suggested in earlier reports.

## Materials and methods

### Animals

12 weeks old C57BL/6J mice were used. They were housed at 24 °C with a 12 h light / dark rhythm.

All mice except the d0 group (the d0 group was designated as untreated) were laser-treated at d0 followed by fundus imaging and angiography. In one eye, large veins were occluded while in the other eye, laser treatment was applied to the retina sparing large vessels as a control. At each of the time points d0, d1, d2, d3, d5, and d8, 18 mice were used for fundus imaging, angiography, and RNA preparation for RNAseq and qPCR, and two additional mice were used for flat mounts stained for hypoxyprobe and Col4 at each time point. Three additional mice were used at d2 for paraffin sections. Five mice were used at d2 or d3 for fundus imaging, angiography, and flatmounts stained for hypoxyprobe and lectin in a preliminary experiment.

All animal procedures adhered to the animal care guidelines of the Institute for Laboratory Animal Research (Guide for the Care and Use of Laboratory Animals) in accordance with the ARVO Statement for the Use of Animals in Ophthalmic and Vision Research and were approved by the local animal welfare committee (Tierschutzkommission).

### Laser treatment

During anesthesia with a mixture of 100 mg ketamine and 5 mg xylazine / kg, mice were injected i. p. with 200 mg/kg of a 100 mg/ml solution of eosin Y in 0.9% NaCl to enhance the laser effect. Eosin Y proved to be effective and has several advantages over rose bengal which was usually applied in other studies. Eosin Y has a higher solubility in water (300 g/l), a high singlet oxygen quantum yield [27], and its absorption maximum at 525 nm is much closer to the wavelength of the irradiating laser (532 nm). In addition, eosin is non-toxic and enabled angiography due to its red fluorescence. The settings of the laser (Visulas 532s, Carl Zeiss Meditec, Jena, Germany) were a power of 50 mW, a spot size of 50 μm, and a duration of 2.5 s. 3–6 laser applications were necessary to occlude a vein as detected by funduscopy. All large veins of one eye of each mouse were occluded while the other eye served as control and received the same laser application scheme (number of laser sites, intensity, and distance from the optic disc) in retinal areas between the large vessels, thus avoiding vascular occlusion.

### Imaging

Fundus images were taken with a Micron III system (Phoenix Research Labs, Pleasanton, CA, USA). Angiography was performed with rhodamine B (100 mg/ml, 200 mg/kg mouse, i.p.) that nicely fits to the TRITC filter of the Micron system, or with fluorescein and a SLO (HRA1, Heidelberg Engineering, Heidelberg, Germany).

Hypoxic retinal areas were visualized by the Hypoxyprobe Red549 Kit (Hypoxyprobe, Burlington, MA, USA). In brief, pimonidazol was dissolved at a concentration of 20 mg/ml and used intraperitoneally at 60 mg/kg. After 3 h, mice were perfused with 2% paraformaldehyde. Retinal flatmounts were prepared and stained with an antibody raised against hypoxyprobe followed by an antibody raised against Col4 (1:250, polyclonal, ab6586, Abcam, Cambridge, UK) or by staining with lectin (10 μg/ml FITC-lectin (BSI) from Griffonia simplicifolia, L9381, Sigma, Taufkirchen, Germany) in order to stain vessels.

Paraffin sections were prepared for histological examination by standard methods after formalin fixation, paraffin embedding and staining with hematoxylin and eosin (HE).

### qPCR

Following fundus imaging and rhodamine B angiography, mice were sacrificed by decapitation. Six retinas from 6 mice were combined to one sample. Three replicate retinal tissue samples were obtained at each time point in each treatment group (laser treatment of all veins or equivalent laser treatment between the large vessels). Veins of one eye of each mouse were occluded while the other eye served as control receiving the same laser application scheme in retinal areas between the large vessels. RNA was isolated from the retinas with the RNeasy kit (Qiagen, Hilden, Germany).

qPCR was performed using standard methods. cDNA was generated with Superscript III reverse transcriptase (Invitrogen, Karlsruhe, Germany) and amplified with the SYBR Premix Ex Taq kit (Takara Bio Europe, Saint-Germain-en-Laye, France). The primers used are presented in Table 1. PCR products were checked on agarose gels and by dissociation curve analysis for single bands. The raw data were analyzed in R (http://www.r-project.org) with the qpcR package [28] that starts with fitting a 6 parameter curve to the raw data. Ct values (from the second derivative) and efficiencies were derived from this curve. Significance was determined by a permutation approach in the propagate function of the qpcR package that was more robust than the Monte Carlo simulation or the error propagation of the same function. PCRs with a replication efficiency of < 1.5 were excluded from further analysis.

**Table 1:** Primer.

Symbol	Gene	Genbank no.	Primer A	Primer B	Fragment length (base pairs)
Actb	Actin beta	NM_007393.3	CACCCGCGAGCACAGCTTCTTTG	TGCACATGCCGGAGCCGTTGTC	116
Aldoa	Aldolase A, fuctose-bisphosphate	NM_001177308.1	AGGCAGTGGGAGGCAATATCT	TTCTCCTCGGTGTTCTCGGT	308
Angpt2	Angiopoietin 2	NM_007426.4	ACCTTACAGGACTCACGGGG	TCATGGTTGTGGCCTTGAGC	264
Ccl2	Chemokine (C-C motif) ligand 2	NM_011333.3	CTGTCATGCTTCTGGGCCTG	AGGCATCACAGTCCGAGTCA	459
Egr1	Early growth response 1	NM_007913.5	GCCGAGCGAACAACCCTATG	ATTGGTCATGCTCACGAGGC	226
Eno1	Enolase 1	NM_023119.2	TCTTTCCTTGCTTTGCAGCGA	CCAGAGCAGGCGCAATAGTT	274
Glut1 (Slc2a1)	Solute carrier family 2 (facilitated glucose transporter), member 1	NM_011400.3	CTGGCGGGAGACGCATAGTT	CAAAGCCAGTAGTCAGGCCG	499
Glut3 (Slc2a3)	Solute carrier family 2 (facilitated glucose transporter), member 3	NM_011401.4	AGGTCACTGAATTCCTGGGGT	TCAGCAGTCCCTCACTTGGT	412
Hif1a	Hypoxia inducible factor 1, alpha subunit	NM_010431.2	GCGAGAACGAGAAGAAAAAGATGAG	CCCTTTTCTCACTGGGCCAT	458
Hif2a (Epas1)	Endothelial PAS domain protein 1	NM_010137.3	AAGTGCACGGTCACCAACAG	AGGTTGCGGGGGTTGTAGAT	479
Igfbp3	Insulin-like growth factor binding protein 3	NM_008343.2	AGCCTAAGCACCTACCTCCC	CTTGGAATCGGTCACTCGGT	126
Il1b	Interleukin 1 beta	NM_008361.3	CTGCTGGTGTGTGACGTTCCCA	AGGGTGGGTGTGCCGTCTTTC	254
Il6	Interleukin 6	NM_031168.1	CCGGAGAGGAGACTTCACAGAGGA	TGGATGGTCTTGGTCCTTAGCCAC	483
Ldha	Lactate dehydrogenase A	NM_010699.2	TCCATTTAAGGCCCCGCCC	AGCACCAACCCCAACAACTG	222
Met	MET proto-oncogene	NM_008591.2	CTGGAGGACAAGACCACCGA	GGCCGTGTAGGACGACATTC	262
Mmp3	Matrix metallopeptidase 3	NM_010809.1	ATGGGCCTGGAACAGTCTTG	AGTCCTGAGAGATTTGCGCC	440
Mmp9	Matrix metallopeptidase 9	NM_013599.3	CTTCCCCAAAGACCTGAAAACCT	GGAAAGGCGTGTGCCAGAAG	489
Nrp2	Neuropilin 2	NM_001077403.1	ACTCCTTTGGGTCATCCGTG	ATCCTCACCTGCAAAAGCTGAT	211
Pfkl	Phosphofructokinase, liver	NM_008826.4	TTTTGGAGGTGATGGGACGG	ATAGGCTTTCCATGCCGGTC	211
Pgk1	Phosphoglycerate kinase 1	NM_008828.2	CATCTCCGGGCCTTTCGACCTCA	TCAGGCATGGGAACACCATCAGGC	257
Pkm	Pyruvat kinase, muscle	NM_001253883.1	GTCACTCCACAGACCTCATGG	TACAAGCGTTGCTGGCCTAA	466
Ppbp (Cxcl7)	Pro-platelet basic protein, Chemokine (C-X-C motif) Ligand 7	NM_023785.2	CAGCCTCACGTTGTTCCCT	TCAAACCCTCAACCCTTCCTG	457
Tgfb1	Transforming growth factor, beta 1	NM_011577.1	CCGCGTGCTAATGGTGGACCG	GCCCTGTATTCCGTCTCCTTGGTT	322
Tgfb2	Transforming growth factor, beta 2	NM_009367.3	TGGCCGAGCAGCGGATTGAACT	ACGTCGAAGGAGAGCCATTCACCC	127
Tgfb3	Transforming growth factor, beta 3	NM_009368.3	GCCAACTTCTGCTCAGGCCC	GCCTCTAGGGTGAGGTCTGTCG	305
Timp4	Timp metallopeptidase inhibitor 4	NM_080639.3	TAAAGGGTTCGAGAAGGCCAAG	CAGAGACACTCATTGGGGGC	291
Tnf	Tumor necrosis factor	NM_013693.2	ATCCGCGACGTGGAACTGGC	TCGGGGCAGCCTTGTCCCTT	431
Vegfa	Vascular endothelial growth factor A	NM_009505.4	CGGGCCTCGGTTCCAGAAGG	TCGGACGGCAGTAGCTTCGC	280
Vegfr1 (Flt1)	Fms-related tyrosine kinase 1	NM_010228.3	CAAGAGCGATGTGTGGTCCT	TGGAGTTCGGTGAAAGCTCC	473
Vim	Vimentin	NM_011701.4	GCTGCTGGAAGGCGAGGAGA	TTCTTGCTGGTACTGCACTGTTGC	238

Primer pairs used for qPCR.

### RNAseq

The same RNA samples that were used for qPCR were used for RNAseq. Library preparation and RNAseq were carried out as described in the Illumina TruSeq Stranded mRNA Sample Preparation Guide, the Illumina HiSeq 1000 System User Guide (Illumina, Inc., San Diego, CA, USA), and the KAPA Library Quantification Kit—Illumina/ABI Prism User Guide (Kapa Biosystems, Inc., Woburn, MA, USA). In brief, 300 ng of total RNA was used for purifying the poly-A containing mRNA molecules using poly-T oligo-attached magnetic beads. Following purification, the mRNA was fragmented to an average insert size of 200–400 bases using divalent cations under elevated temperature (94 °C for 4 min). The cleaved RNA fragments were copied into first strand cDNA using reverse transcriptase and random primers. Strand specificity was achieved by replacing dTTP with dUTP in the Second Strand Marking Mix (SMM), followed by second strand cDNA synthesis using DNA Polymerase I and RNase H. The incorporation of dUTP in second strand synthesis quenches the second strand during amplification, because the polymerase used in the assay is not incorporated past this nucleotide. The addition of Actinomycin D to First Strand Synthesis Act D mix (FSA) prevents spurious DNA-dependent synthesis, while allowing RNA-dependent synthesis, improving strand specificity. These cDNA fragments then had the addition of a single 'A' base and subsequent ligation of the adapter. The products were purified and enriched with PCR to create the final cDNA library. The libraries were quantified using the KAPA SYBR FAST ABI Prism Library Quantification Kit (Kapa Biosystems, Inc., Woburn, MA, USA). Equimolar amounts of each library were pooled, and the pools were used for cluster generation on the cBot with the Illumina TruSeq SR Cluster Kit v3. The sequencing run was performed on an HiSeq 1000 instrument using the indexed, 50 cycles single-read (SR) protocol and the TruSeq SBS v3 Reagents according to the Illumina HiSeq 1000 System User Guide. Image analysis and base calling resulted in .bcl files, which were converted into .fastq files with the CASAVA1.8.2 software. Library preparation and RNAseq were performed at the service facility “KFB—Center of Excellence for Fluorescent Bioanalytics” (Regensburg, Germany; www.kfb-regensburg.de).

Data were evaluated in R with packages from Bioconductor (http://bioconductor.org). Reads were mapped to the mouse genome (Gencode_vM11) with STAR 2.5.2b (https://github.com/alexdobin/STAR) [29]. Features were counted with the featureCount function of the Rsubread package (Subread 1.5.1 for R [30]), and differential gene expression was analyzed with DESeq2 1.14.1 [31]. DESeq2 applies a set of sophisticated statistical tools including normalisation between samples, empirical Bayes shrinkage for dispersion estimation and for fold-change estimation, and correction for multiple testing. Gene ontology analysis was done with debrowser 1.2.4.2 and GO.db 3.4.0. The data are available under the accession number GSE101398 at https://www.ncbi.nlm.nih.gov/geo/query/acc.cgi?acc=GSE101398.

## Results

### Retinal vein occlusion

**Fig 1:**
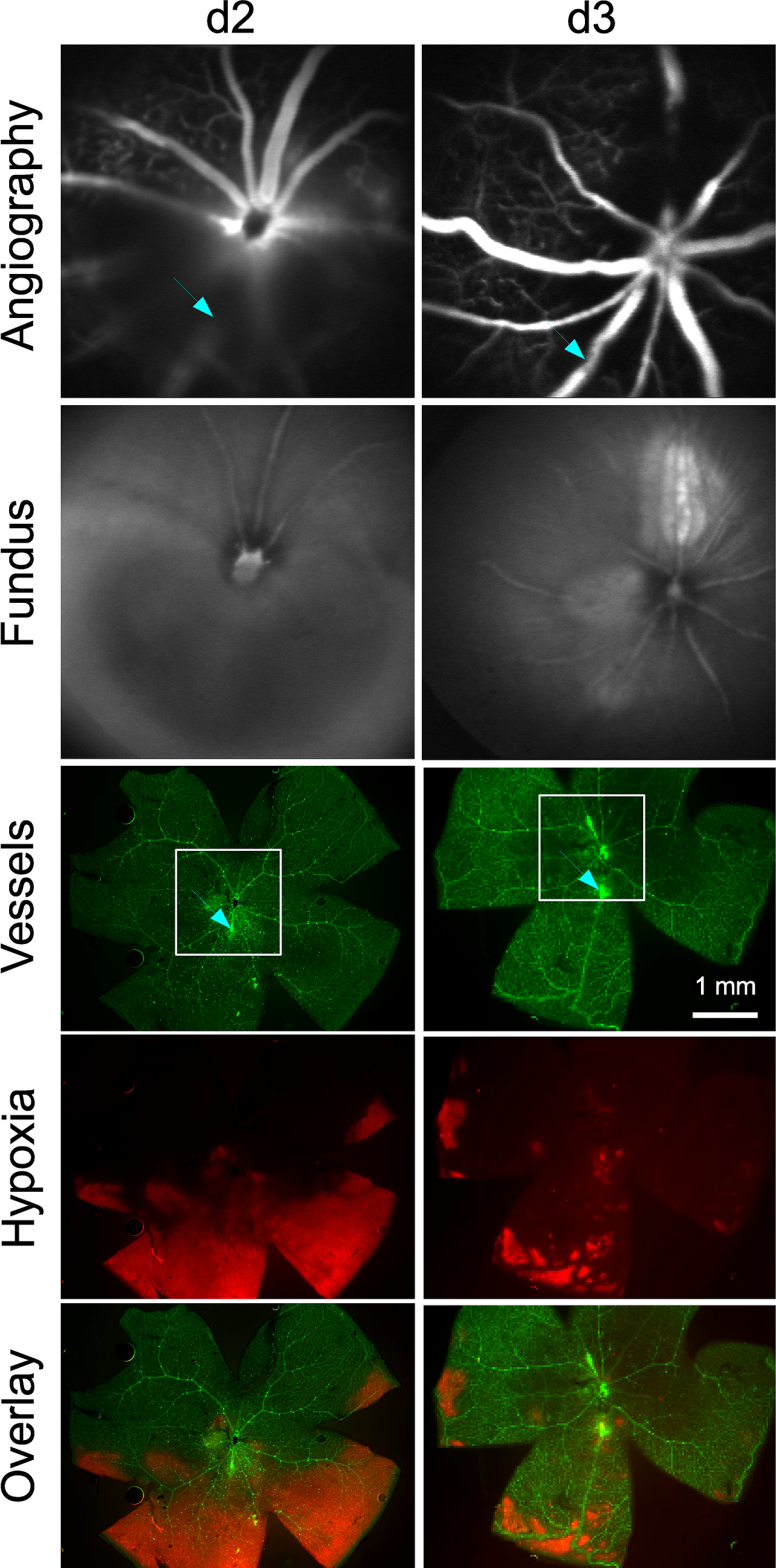
RVO and hypoxic area. In a preliminary experiment, two veins were occluded in two mice at d0. Fluorescein angiography by SLO showed the occlusion of the veins, and fundus images taken by SLO are shown for comparison. Retinal flatmounts were stained for vessels with lectin (green) and with hypoxyprobe (red) for hypoxic areas. The squares show the positions of the SLO images while the arrows show the position of laser sites. In one mouse at d2, the vein to the left was re-opened while the lower vein was still closed (arrow) and surrounded by a hypoxic area. In the other mouse at d3, the lower vein was re-opened (arrow) but showed some remnants of the hypoxic area. Typical results from a study of 5 mice are shown.

**Fig 2:**
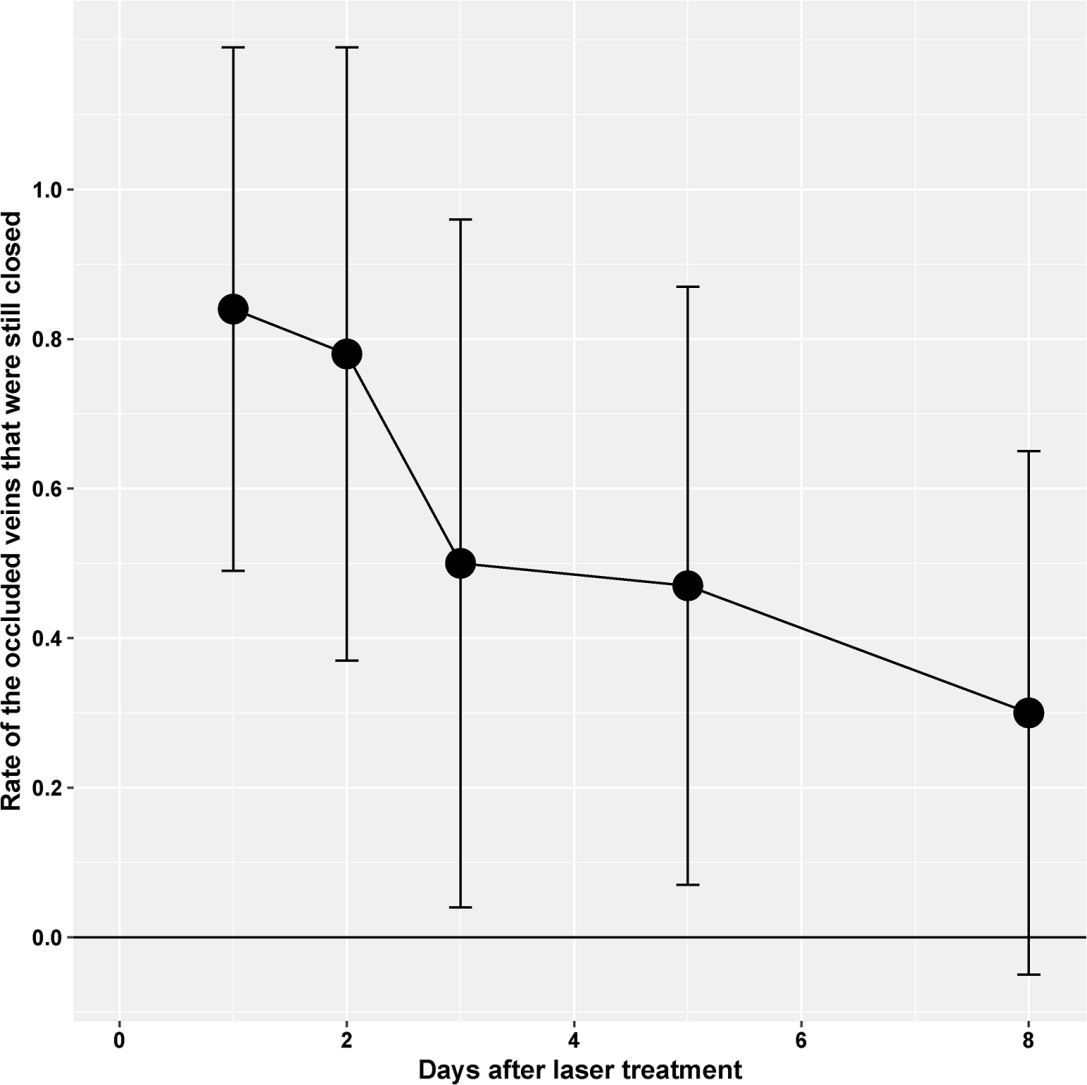
Time course of occlusion. RVO was evaluated in fundus images and by angiography. The diagram shows the mean rate of occluded veins that were still closed at the respective day. Veins were assumed to be occluded if there was a visible thrombus, an interruption in blood flow, a difference in vessel caliber (central constriction, peripheral dilation), leakage, tortuositas, or neovascularization. Half of the veins were re-opened after three days. Error bars indicate standard deviation. The values are from 15–17 retinae of the same animals used for the qPCR and RNAseq experiments.

Retinal veins were occluded by laser treatment after systemic application of eosin Y as a photosensitizer. The occlusion was visible during slit lamp laser treatment as the vein became narrow at the laser site and blood flow subsided. Subsequent angiography revealed leaking vessels at the laser sites. Two days after laser treatment, the retinal area distal from the treatment site showed reduced perfusion and hypoxic areas as detected by hypoxyprobe staining (Fig 1). A time course analysis of vessel occlusion showed that half of the vessels stayed closed for at least three days (Fig 2). Around one third of the veins were still unperfused after eight days.

**Fig 3:**
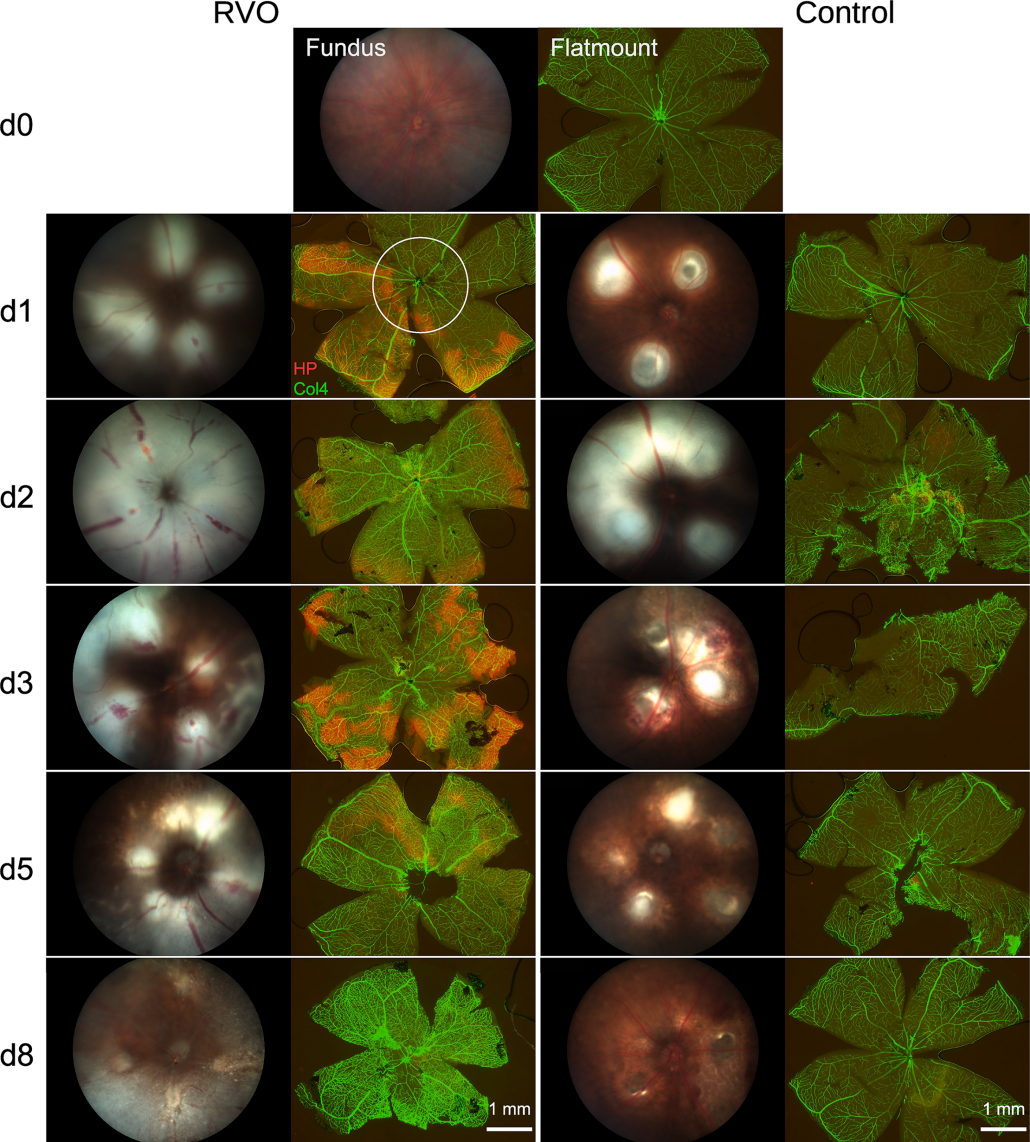
Time course of RVO and hypoxia. RVO was induced at d0 in two mice per time point. Fundus images were taken by a Micron III camera and flatmounts were prepared at the indicated days. Flatmounts were stained for vessels with an antibody raised against collagen IV (Col4, green) and with hypoxyprobe (red) for hypoxic areas. Flatmounts at d0 were not laser-treated. In one eye of each mouse, the veins were occluded (RVO) while the other eye was laser-treated between the large vessels (control). The circle indicates the area of the corresponding fundus image. Hypoxic areas were found from d1 to d3, diminished at d5 and disappeared completely at d8. No hypoxia staining was observed after intervenous laser treatment in the control group. The large edematous areas (gray areas of the fundus images) at the laser sites were observed both in the RVO and the control group. They resulted in weaker hypoxia staining (compare the RVO eye at d2).

**Fig 4:**
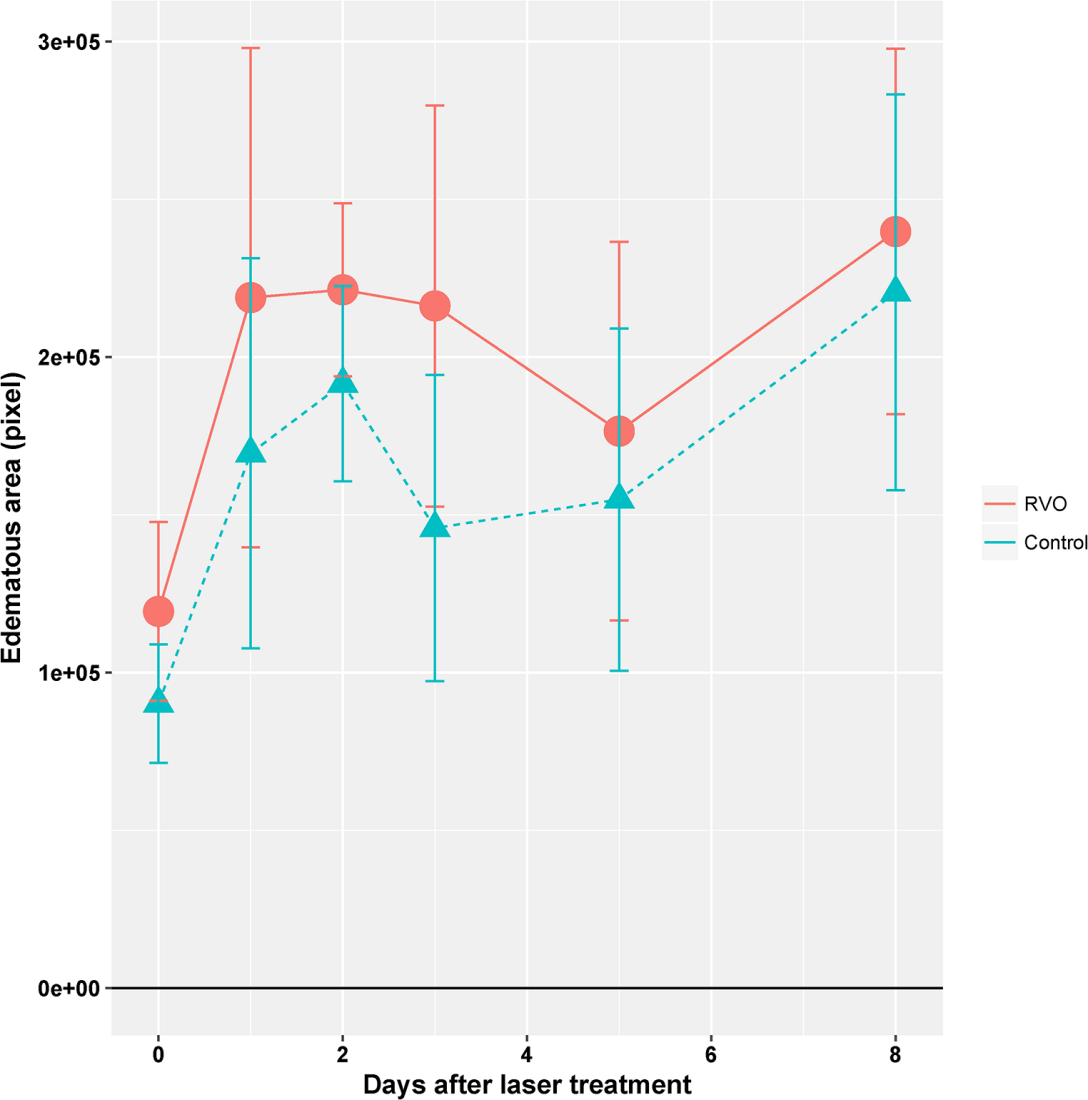
Time course of edema formation. The edematous area of the retina in fundus images like those shown in Fig x2 was evaluated. Edema is the gray area that contrasts with the brown intact retina. Though not significant, the edematous area was somewhat larger in RVO than in the control laser treatments. It increased from d0 to d1. Error bars indicate standard deviation. The values are from 11–17 retinae of the same animals used for the qPCR and RNAseq experiments. The increase at d8 is an artifact.

At funduscopic examination, all laser-treated retinas showed large whitish edematous areas at the laser sites (Fig 3), irrespective of laser targeting. These areas were somewhat more extended in RVO eyes as compared to controls, but the difference was not statistically significant. (Fig 3 and Fig 4).

**Fig 5:**
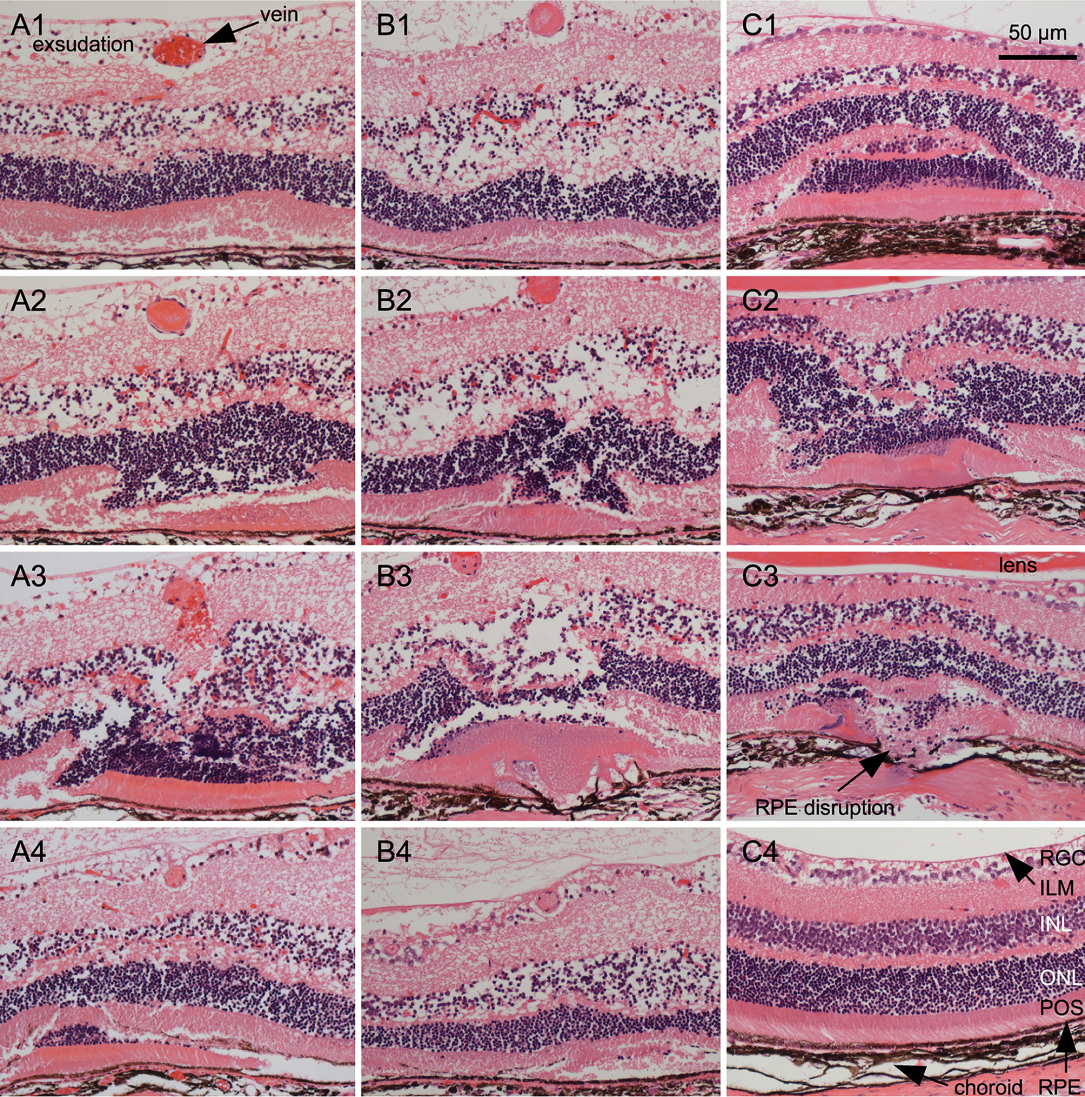
Histology of the occlusion site. Veins were occluded at d0. Two days later, eyes were prepared for serial paraffin sections that were stained with HE. Typical sections from three RVO eyes and three control eyes are shown. A1—A4: Sections at different positions of a single laser site. The RPE is slightly affected and Bruch’s membrane is intact. The inner limiting membrane is detached by a large inner retinal serous exsudation, and the INL is also affected by a serous exsudation. A1 is from the distal part of the laser site with the vein containing erythrocytes. A2 shows a fold (invagination) within the outer retina and a vein that contains a fibrous plug. A3 is from the center of the laser site showing a large retinal invagination and a bleeding vein containing a fibrous plug. A4 is from the proximal part of the laser site showing the retinal fold within the RPE and a decreased vein. The whole series of sections is shown in the supplemental S1 Fig. B1—B4: Sections at different positions of another single laser site. The inner limiting membrane is detached by a large inner retinal serous exsudation, and the INL is also affected by a serous exsudation. RPE and Bruch’s membrane are perforated as is seen in B3. B1 shows the distal part of the laser site with a fold of photoreceptor outer segments within the RPE and a vein containing a fibrous plug. B2 shows a fold within the outer retina. B3 is from the central part of the laser site showing a retinal fold and fused material of photoreceptors, RPE, and choroid. B4 shows the proximal vein that is diminished and a lateral part of the laser site with a fold of outer segments of the photoreceptors. The retina shows unequal thickness. The whole series of sections is shown in the supplemental S2 Fig. C1—C3: Sections from control laser sites without vein occlusion. The morphology of the laser sites is principally equal to RVO laser sites. C1 shows a fold of the outer retina of a laser site with intact Bruch’s membrane. The choroid is affected as shown by its reduced thickness at the center of the laser site. C2 and C3 are sections from a laser site with disrupted RPE and choroid. The choroid shows a reduced thickness, and even the sclera is affected. The material of the outer retinal fold is partially fused. C4 is a section from an intact retina for comparison. ILM: inner limiting membrane; INL: inner nuclear layer; ONL: outer nuclear layer; POS: photoreceptor outer segments; RGC: retinal ganglion cells.

Examination of laser sites in serial paraffin sections stained with hematoxylin and eosin (HE) showed damage in outer retinal layers including photoreceptor outer segments and retinal pigment epithelium and, in severe cases, even choroid and sclera (RPE, Fig 5 and supplemental S1 and S2 Fig that show serial sections of two laser sites). Typically, a large fold was generated from parts of the outer retina. The inner retinal layers appeared to be less affected. In addition, serous exsudation was observed within the retinal ganglion cell layer showing detachment of the inner limiting membrane which fits the angiographic and funduscopic images. The inner nuclear layer showed serous exsudation, too. The defects of ocular tissues generated by laser treatment were similar in RVO and in control laser treatments. In cross sections of the vein, a fibrous plug generated by the laser treatment after injection of a photosensitizer was visible. The proximal part of the vein was much smaller than the distal part.

### Hypoxia time course

Hypoxia was detected by hypoxyprobe staining in all RVO eyes from d1 to d5 and disappeared afterwards (Fig 3). No hypoxia was found in the control eyes following retinal laser treatment sparing large vessels. While the hypoxic areas in RVO eyes were rather homogeneous during the first days, they became disjunct during reperfusion so that increasingly smaller hypoxic areas remained within the capillary regions until hypoxia was no longer detectable.

### Gene expression profiling

**Fig 6:**
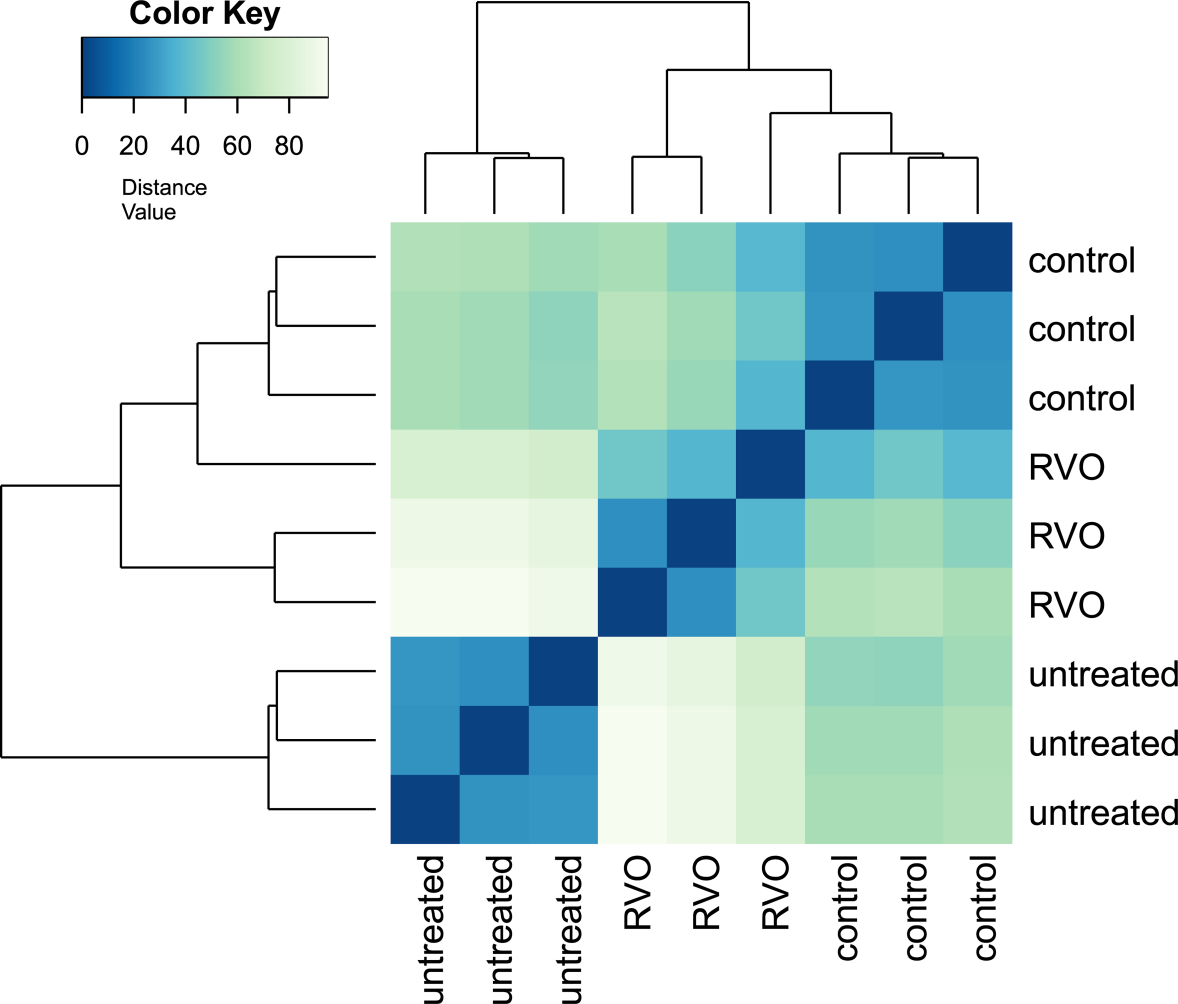
Sample-to-sample distances.

Next, we investigated the effect of hypoxia on global gene expression patterns by RNAseq at d2. RVO eyes were compared to control laser-treated eyes as well as to untreated controls. There were large differences between RVO eyes and untreated controls, as well as between control laser-treated eyes and untreated controls. The differences between RVO eyes and control laser-treated eyes were much smaller (Fig 6). Similarly, while the maximal fold change was 175 for comparisons between RVO and untreated eyes it was only 8 for comparisons between RVO and control laser treatment (Table 2). This indicates that it is important to use an appropriate laser-treated control group.

**Table 2:** Genes up-regulated in RVO versus laser-treated control.

**A: RVO versus control**							
**ID**	**treated**	**treated**	**treated**	**control**	**control**	**control**	**Fold change**
Car9	247.06	139.22	180.34	19.09	16.71	13.76	8.36
Csn3	175.79	94.50	123.65	18.99	8.00	7.84	7.31
Adm	857.16	549.88	593.18	55.37	88.35	96.68	6.96
Ecel1	339.52	287.59	286.47	52.83	65.50	34.48	5.32
C3	3752.94	1903.99	2880.83	452.78	506.02	476.76	5.27
Plaur	812.58	245.02	543.72	85.41	81.83	59.16	5.14
Siglec15	67.80	21.35	26.20	1.79	3.52	0.95	5.00
Serpine1	2081.16	1030.78	1770.51	262.49	359.53	213.17	4.96
Hsd17b2	1244.57	818.29	1174.96	222.67	177.31	212.19	4.87
Car6	115.14	65.02	62.91	11.67	13.36	11.79	4.68
Fam179a	410.80	317.09	336.90	81.67	73.11	55.22	4.66
Ndufa4l2	549.26	506.09	281.48	72.15	69.89	101.62	4.61
Neurog2	478.71	487.85	563.64	126.22	100.25	89.77	4.59
Slc13a4	828.14	655.61	754.89	142.00	176.17	147.03	4.57
Myc	1392.00	487.98	1085.65	191.38	189.22	139.13	4.55
Mylk2	100.24	79.26	140.22	24.87	18.81	6.85	4.52
Mmp3	232.90	222.57	342.92	61.17	60.07	42.38	4.32
Chil1	17888.23	11158.72	13730.36	3380.27	3058.64	3027.78	4.25
Crlf1	187.17	206.27	232.80	40.72	54.61	40.41	4.22
Tuba1c	781.65	476.75	860.79	186.42	154.49	104.57	4.14
Tarm1	36.00	15.25	32.31	2.93	3.48	1.94	4.10
Slpi	93.94	67.04	66.01	17.59	17.66	4.88	4.07
Apln	672.08	378.15	728.45	139.51	115.45	132.22	4.06
Cyb5r2	144.52	96.51	94.37	15.37	35.00	9.81	3.98
Lif	1711.80	1146.64	1729.56	405.64	380.13	296.10	3.94
Egln3	1122.19	714.57	569.25	157.66	192.45	193.43	3.92
Rin1	592.95	235.85	414.60	84.14	90.47	91.75	3.90
Hmox1	17733.97	10204.15	11834.41	1557.16	2697.39	545.86	3.89
Tnfrsf12a	3747.66	1399.83	3264.12	641.75	656.77	525.14	3.88
Clec4d	203.01	102.68	213.79	48.97	34.03	15.73	3.88
Derl3	600.47	107.82	417.55	39.66	39.52	29.55	3.88
H2-T-ps	60.12	29.46	31.43	7.83	4.62	5.88	3.87
Fam83g	143.05	39.68	115.14	12.93	24.19	6.85	3.86
Tcstv1	66.13	45.73	80.49	16.32	9.01	11.80	3.86
Akr1b8	703.02	407.62	592.08	156.30	111.11	134.20	3.83
Cdkn1a	2345.95	949.48	1631.99	338.31	458.21	314.86	3.79
Tubb6	2719.50	1605.12	2258.00	611.55	548.28	474.79	3.74
Gm6634	48.41	13.24	33.40	4.17	2.41	3.91	3.73
Clec4n	152.21	82.35	200.05	31.00	39.39	20.67	3.69
Ccnd1	6253.00	3845.62	6324.98	1621.91	1376.02	1090.82	3.69
Avil	287.10	169.73	190.99	59.88	48.13	48.31	3.66
Gm44658	62.52	48.72	35.65	13.80	1.37	7.85	3.65
Krtap4-16	91.83	9.20	46.00	5.51	4.63	0.95	3.65
Cdh23	70.36	38.62	62.75	10.36	8.99	14.77	3.63
Krt24	130.11	43.74	114.10	20.09	20.95	14.75	3.63
Runx1	639.08	258.22	498.57	121.39	128.40	70.02	3.58
Stc2	799.94	358.84	486.13	108.27	157.69	136.18	3.52
Cxcl1	204.15	72.21	215.81	16.67	53.45	11.78	3.52
Serpinb1a	110.41	192.96	107.04	36.94	33.99	27.58	3.52
Fbln2	480.16	474.65	508.07	122.62	171.73	94.71	3.51
**B: RVO versus untreated**							
**ID**	**treated**	**treated**	**treated**	**untreated**	**untreated**	**untreated**	**Fold change**
Bcl3	1518.62	1027.59	1344.66	8.76	4.02	9.47	175.64
Timp1	807.11	721.01	811.04	3.37	5.32	6.54	150.51
S100a9	682.27	240.24	692.09	5.14	1.34	2.05	145.90
Ccl2	422.44	285.70	450.19	5.07	2.65	1.99	112.84
Lcn2	10449.02	4804.19	9161.28	67.25	80.19	90.31	98.06
Hmox1	13670.02	7363.17	8627.07	99.87	120.16	93.17	93.33
Mmp3	181.51	161.99	253.67	2.89	1.20	1.82	85.05
Lgals3	3501.21	2664.98	3053.87	60.55	32.49	28.29	81.38
Plaur	631.87	176.39	398.78	6.89	2.66	5.03	70.85
Edn2	3169.89	2282.56	2935.51	41.61	40.25	51.45	66.11
Egr2	192.63	126.14	233.68	2.85	1.18	3.38	65.75
S100a8	519.89	243.62	588.78	3.24	5.30	2.02	65.66
Sprr1a	198.93	86.97	129.46	2.67	1.12	1.69	58.34
Il1rn	384.72	147.26	375.42	6.83	3.96	3.49	58.07
Arg1	810.81	408.21	811.04	12.27	11.84	10.94	57.20
Lif	1323.10	828.41	1263.84	17.50	20.91	24.03	56.10
Cxcr2	150.89	79.61	150.37	2.61	1.09	1.66	55.55
Clec4d	159.56	74.23	158.94	2.63	1.10	1.67	55.40
Flnc	1161.41	587.10	1240.83	17.51	11.83	24.04	55.02
Socs3	3324.24	2427.08	3074.79	60.56	51.87	61.56	53.87
Gm23935	267.13	388.95	177.34	4.98	6.58	3.47	52.22
Serpina3n	9863.45	17284.59	11436.61	316.08	209.13	251.78	50.42
Pf4	131.00	78.63	102.73	2.47	1.04	1.58	48.73
Nlrc5	482.06	362.05	589.11	5.12	7.92	16.78	48.54
Cxcl1	160.64	51.64	160.77	2.60	1.09	1.65	48.35
Clec4n	119.75	59.45	149.47	2.51	1.06	1.60	46.68
Socs1	127.27	86.53	153.68	4.61	1.09	1.65	46.25
Ccl7	101.24	70.38	123.67	2.43	1.03	1.55	45.86
Prss56	4506.07	2723.15	5150.50	79.42	106.06	87.51	45.36
Lad1	1623.00	889.11	1310.85	31.37	37.72	18.21	44.03
Cebpd	4566.62	3474.70	3756.37	106.94	104.77	86.07	42.15
Hsd17b2	962.97	591.52	859.33	22.74	19.62	18.23	41.93
9330175E14Rik	98.93	73.74	178.54	2.56	1.07	3.24	41.47
Slfn1	141.93	58.68	129.54	2.51	1.06	3.22	39.43
Car9	193.75	101.23	133.26	6.54	1.13	3.31	39.33
Cdsn	900.54	793.40	620.66	15.77	15.73	31.30	38.86
Upp1	477.99	155.31	326.86	8.64	6.60	7.97	38.76
Il1b	168.50	77.72	198.59	2.72	2.52	4.84	38.50
Hmga2	1341.84	364.54	1272.44	10.51	10.53	19.68	37.56
Crlf1	146.19	150.84	172.29	4.75	5.20	3.34	36.74
Tuba1c	605.90	344.62	631.01	14.01	14.44	16.78	35.84
Serpine1	1608.33	744.15	1293.41	34.83	26.08	41.41	35.79
C3	2896.83	1374.33	2102.21	67.51	59.65	57.26	34.94
Junb	6160.20	4326.56	5211.85	168.71	182.13	132.21	34.11
Chil1	13788.60	8051.83	10009.14	340.15	345.82	296.51	33.48
Slfn4	312.47	90.23	371.02	3.02	1.25	1.90	33.23
Slc6a2	470.50	203.44	369.16	12.21	10.52	9.45	32.55
Csn3	138.57	68.80	91.71	4.48	1.03	3.18	32.33
Lilrb4a	144.17	88.67	150.28	2.62	2.49	6.28	32.18
Ccl3	94.12	54.78	92.44	2.27	2.38	1.46	32.01

The 50 genes with the highest fold change in the group with RVO laser treatment at d2 compared to the group with control laser treatment at d2 (A) and the 50 genes with the highest fold change in the group with RVO laser treatment at d2 compared to the untreated group (B) as determined by RNAseq. Values are mean counts, pAdjust < 0.01.

To gain further insight into possible functional implications of the gene expression alterations, a gene ontology analysis was performed (Table 3). The most significantly affected ontology groups in the comparison of RVO and untreated eyes were related to inflammation or wounding. In contrast, in the comparison of RVO and control laser-treated eyes, some ontology groups related to angiogenesis were affected. Ontology groups related to hypoxia did not show up among the first 100 groups with the highest significance score.

**Table 3:** The 50 most significant GO terms.

**A: RVO versus control**	
**ID**	**Description**
GO:0001525	*angiogenesis*
GO:0045765	*regulation of angiogenesis*
GO:1901342	*regulation of vasculature development*
GO:0009611	response to wounding
GO:0045766	*positive regulation of angiogenesis*
GO:1904018	*positive regulation of vasculature development*
GO:0060326	**cell chemotaxis**
GO:0042060	wound healing
GO:0030593	**neutrophil chemotaxis**
GO:0050900	**leukocyte migration**
GO:1990266	**neutrophil migration**
GO:0030595	**leukocyte chemotaxis**
GO:0071621	**granulocyte chemotaxis**
GO:0048608	reproductive structure development
GO:0061458	reproductive system development
GO:0032103	positive regulation of response to external stimulus
GO:0097530	**granulocyte migration**
GO:0097529	**myeloid leukocyte migration**
GO:0050729	**positive regulation of inflammatory response**
GO:0070098	**chemokine-mediated signaling pathway**
GO:0002548	**monocyte chemotaxis**
GO:0002687	**positive regulation of leukocyte migration**
GO:0070372	regulation of ERK1 and ERK2 cascade
GO:0070371	ERK1 and ERK2 cascade
GO:0071347	**cellular response to interleukin-1**
GO:0071674	**mononuclear cell migration**
GO:0031349	positive regulation of defense response
GO:0001890	placenta development
GO:0002685	**regulation of leukocyte migration**
GO:0030335	positive regulation of cell migration
GO:2000147	positive regulation of cell motility
GO:0002690	**positive regulation of leukocyte chemotaxis**
GO:0051272	positive regulation of cellular component movement
GO:0040017	positive regulation of locomotion
GO:2001233	regulation of apoptotic signaling pathway
GO:0070555	**response to interleukin-1**
GO:2001237	negative regulation of extrinsic apoptotic signaling pathway
GO:0002688	**regulation of leukocyte chemotaxis**
GO:2001234	negative regulation of apoptotic signaling pathway
GO:0048245	**eosinophil chemotaxis**
GO:2001236	regulation of extrinsic apoptotic signaling pathway
GO:0031099	regeneration
GO:0097191	extrinsic apoptotic signaling pathway
GO:0019221	**cytokine-mediated signaling pathway**
GO:0050679	positive regulation of epithelial cell proliferation
GO:0071345	**cellular response to cytokine stimulus**
GO:0070374	positive regulation of ERK1 and ERK2 cascade
GO:0072216	positive regulation of metanephros development
GO:0050878	regulation of body fluid levels
GO:0050921	**positive regulation of chemotaxis**
**B: RVO versus untreated**	
**ID**	**Description**
GO:0071345	**cellular response to cytokine stimulus**
GO:0050900	**leukocyte migration**
GO:0030335	positive regulation of cell migration
GO:0051272	positive regulation of cellular component movement
GO:2000147	positive regulation of cell motility
GO:0040017	positive regulation of locomotion
GO:0060326	**cell chemotaxis**
GO:0001525	*angiogenesis*
GO:0032103	positive regulation of response to external stimulus
GO:0097529	**myeloid leukocyte migration**
GO:0030595	**leukocyte chemotaxis**
GO:0019221	**cytokine-mediated signaling pathway**
GO:0031349	positive regulation of defense response
GO:0002685	**regulation of leukocyte migration**
GO:0001819	**positive regulation of cytokine production**
GO:0002237	response to molecule of bacterial origin
GO:0050727	**regulation of inflammatory response**
GO:0002687	**positive regulation of leukocyte migration**
GO:0045765	*regulation of angiogenesis*
GO:0071621	**granulocyte chemotaxis**
GO:0009611	response to wounding
GO:1901342	*regulation of vasculature development*
GO:0050778	**positive regulation of immune response**
GO:0032496	response to lipopolysaccharide
GO:0097530	**granulocyte migration**
GO:0045785	positive regulation of cell adhesion
GO:0002443	**leukocyte mediated immunity**
GO:0002460	**adaptive immune response based on somatic recombination of immune receptors built from immunoglobulin superfamily domains**
GO:0002250	**adaptive immune response**
GO:1903706	regulation of hemopoiesis
GO:0070371	ERK1 and ERK2 cascade
GO:0002683	**negative regulation of immune system process**
GO:0030593	**neutrophil chemotaxis**
GO:0034341	response to interferon-gamma
GO:0050920	**regulation of chemotaxis**
GO:1990266	**neutrophil migration**
GO:0007159	**leukocyte cell-cell adhesion**
GO:0042060	wound healing
GO:0045088	**regulation of innate immune response**
GO:0070663	**regulation of leukocyte proliferation**
GO:0043410	positive regulation of MAPK cascade
GO:1902105	**regulation of leukocyte differentiation**
GO:0050679	positive regulation of epithelial cell proliferation
GO:0050921	**positive regulation of chemotaxis**
GO:0032944	**regulation of mononuclear cell proliferation**
GO:0070372	regulation of ERK1 and ERK2 cascade
GO:0002690	**positive regulation of leukocyte chemotaxis**
GO:0050670	**regulation of lymphocyte proliferation**
GO:0050730	regulation of peptidyl-tyrosine phosphorylation
GO:0002688	**regulation of leukocyte chemotaxis**

GO analysis using genes with fold change > 3 and pAdjust < 0.01. The 50 most significant GO groups are presented and ordered by significance. GO groups related to angiogenesis are labeled in italics while those related to inflammation are labeled in bold. Note that a group of GO terms related to angiogenesis are highly significant in the comparison of RVO versus controls while the comparison of RVO versus untreated shows almost exclusively GO terms related to inflammation. The first GO group related to hypoxia was found at positions 412 and 468, respectively.

### Time course of gene expression changes

The time course of gene expression from d0 to d8 were investigated by quantitative RT-PCR. A general feature of all kinetics was that RVO and controls ran in parallel. This suggests that the changes in gene expression were related to laser-induced tissue damage and subsequent tissue repair rather than to hypoxia.

**Fig 7:**
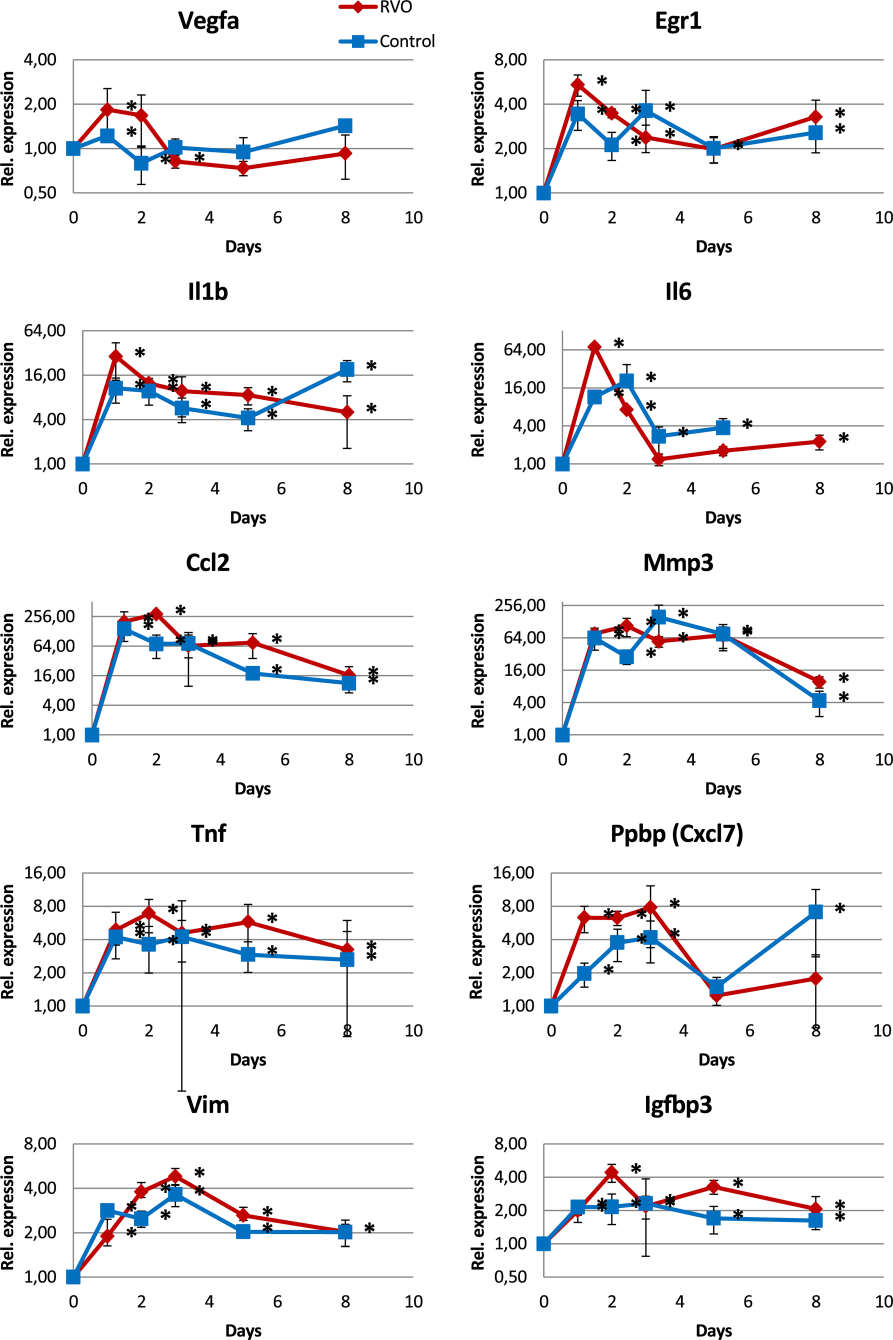
mRNA expression analysis (qPCR) of genes strongly up-regulated in RVO. The first 4 factors had an expression maximum at d1 while the others had a maximum at d2 or d3. All of them are involved in inflammation or responses to stress. Most interestingly, the up-regulation found in RVO was also detected in the controls that were laser-treated between the large vessels. Note the logarithmic expression scale that shows equal distances for factors that are up- or down-regulated. Asterisks at the right of the corresponding data point indicate significant differences as compared to d0. Mice at d0 were not laser-treated.

The most striking result was the increased expression of genes related to inflammation both in the RVO and the control groups as compared to the untreated group at d0 (Fig 7). Factors like Il1b, Il6, or Egr1 peaked at d1 while others like Ccl2, Tnf, Mmp3, Ppbp (Cxcl7), Igfbp3, or Vim peaked at d2 or d3 indicating a strong inflammatory response. Ccl2 was 290-fold increased while Mmp3 was more than 100-fold increased.

**Fig 8:**
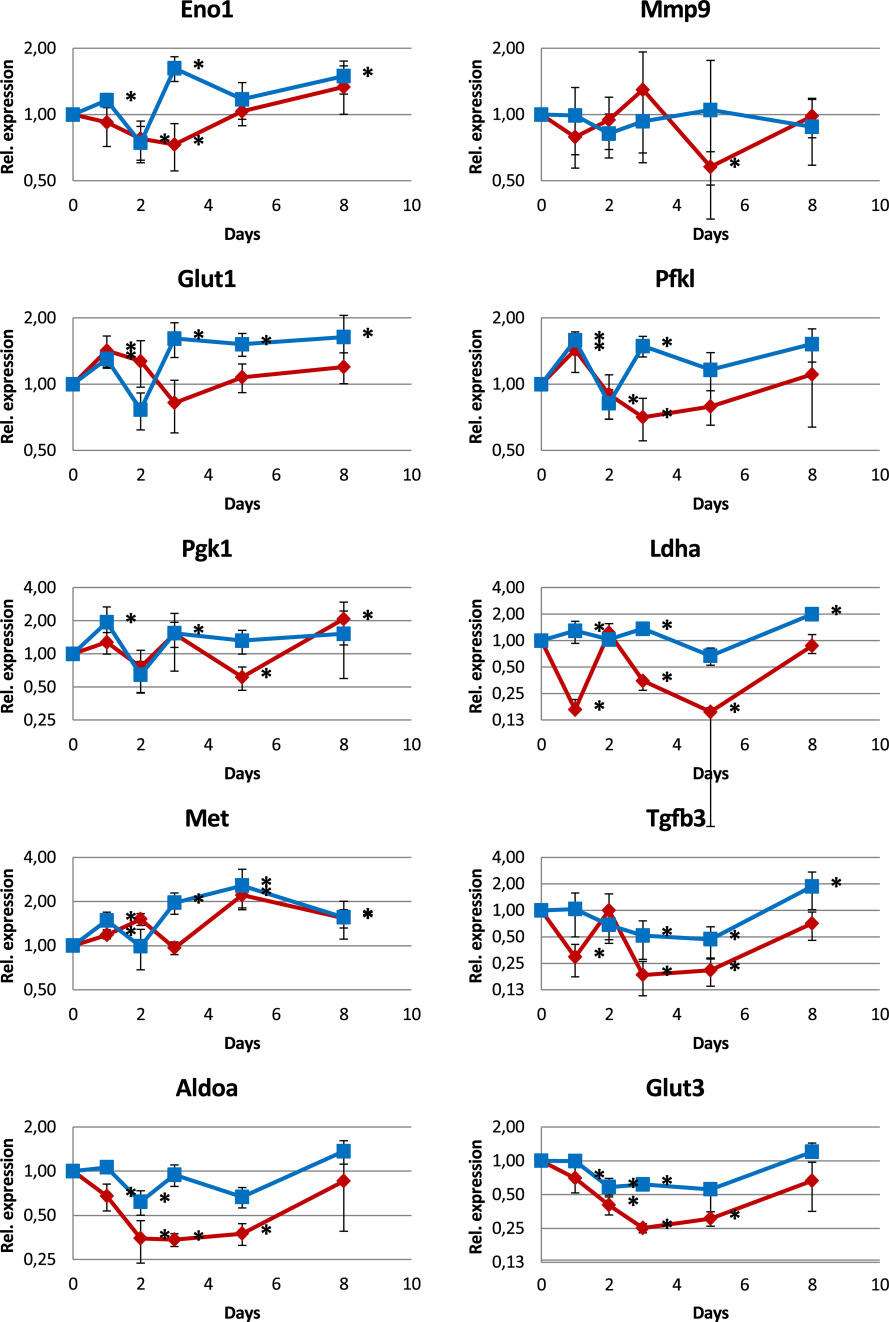
mRNA expression analysis (qPCR) of genes regulated by hypoxia. Most of the genes show only small changes not exceding an up-regulation of a factor of 2 while some show a down-regulation instead.

**Fig 9:**
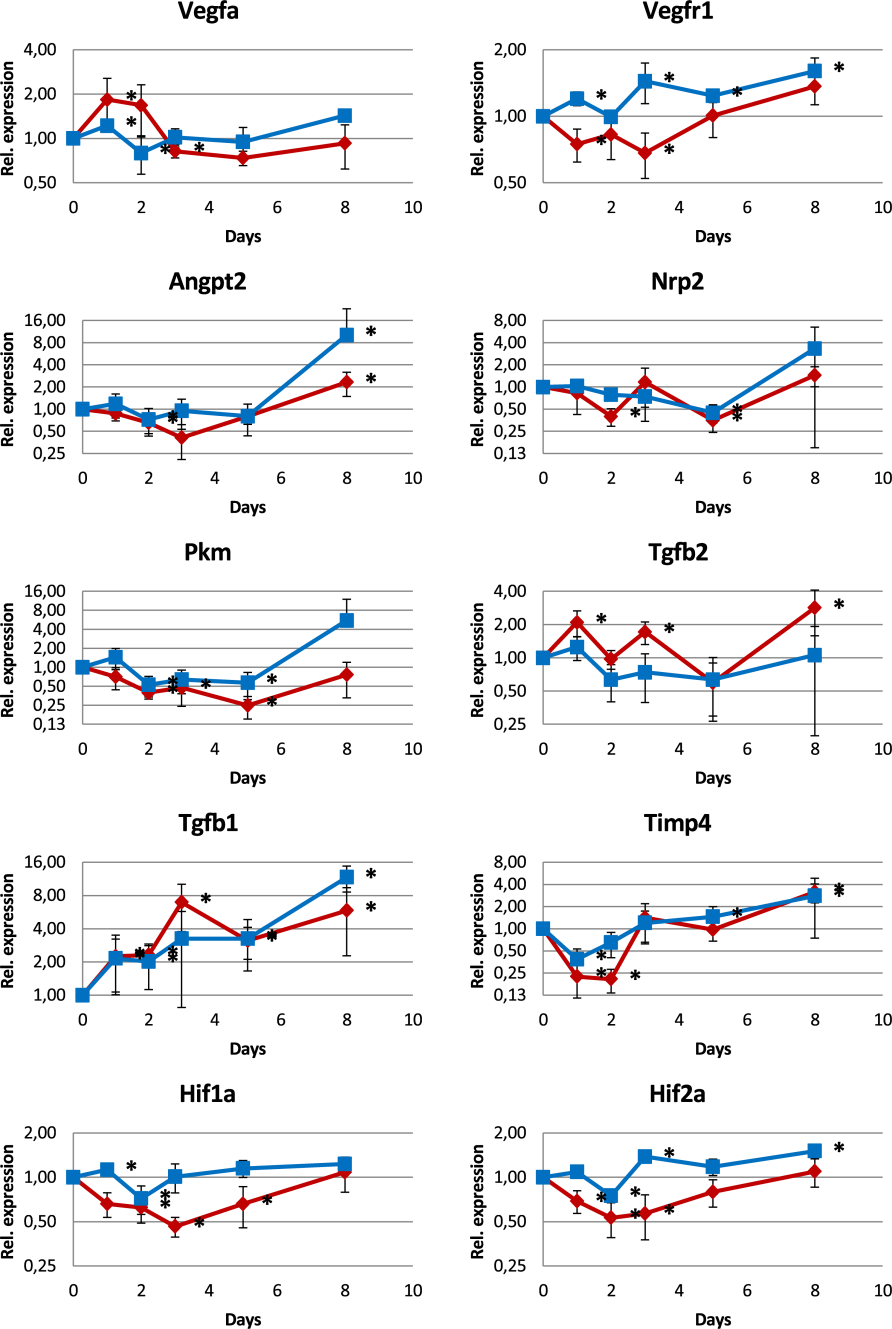
mRNA expression analysis (qPCR) of genes involved in angiogenesis and other processes. The regulation of most of these genes is weak, not exceeding a factor of 2.

In contrast, expression of hypoxia-responsive genes was not increased. Factors like Eno1, Mmp9, Glut1, Pfkl, Pgk1, Pkm, Ldha, Met, Tgfb3, Aldoa, Glut3, and Angpt2 that have hypoxia responsive elements within their promoters, as well as Hif1a and Hif2a were up-regulated not more than 2-fold (Figs 8 and 9). Some of them, Aldoa, Glut3, Tgfb3, and Ldha were down-regulated.

Many factors showed an increased expression at d8 which was in contrast to the reduced expression of inflammatory factors at d8. This may indicate reduced inflammation and increased repair of the damaged tissue.

The factors Vegfa, Vegfr1, Angpt2, and Nrp2 are not only hypoxia responsive genes but also angiogenesis factors. Vegfa showed a peak at d1 and d2, however, the peak was small and its physiological relevance is not clear.

## Discussion

Retinal vein occlusion by laser treatment of retinal veins has been described in several species including pig, rabbit, and rat. But there are only few reports in mice.

We used eosin Y as a photosensitizer and subsequent laser treatment to induce RVO in mice. Our finding of persistent hypoxia for up to 5 days after retinal vein occlusion is in line with other reports [19,25]. Nevertheless, expression of genes typically altered in hypoxia were not upregulated in whole retina samples obtained in the hypoxic period (Fig 7). Instead, an intense inflammatory response dominated the gene expression changes. Earlier reports described similar increases of inflammatory factors like Ccl2, Il1b, Il6, and Tgfb1 [18,21,32,33].

This intense inflammatory response corresponds to the large edematous areas found in fundus images as well as in histological sections. Other studies described these areas as hemorrhage and cystoid edema [21] which are useful for investigating inflammatory processes. The laser treatment additionally induced severe damage to the outer retina, the RPE and often to the choroid at the laser site. Similar destructions including damage of the outer retina were reported earlier [20,21].

The large laser damage and the intense inflammatory response might have obscured angiogenesis- or hypoxia-related gene expression changes. While the size of the laser lesion in our study is comparable to the lesion size of studies with larger animal species like rat or pig, the resulting hypoxic area of the occluded vein is much smaller in mice. That means that the ratio of the area of laser damage to the hypoxic area is disadvantageous in mice and the influence of the damage-induced inflammatory response is much greater than in larger animals rendering mice a rather unattractive model for RVO. Indeed, such an intense inflammatory response was not found in proteomic studies of RVO in pig [10,11]. Our observation might have been missed when using untreated controls only and stresses the importance of appropriate control laser treatment groups.

As a consequence, it would be desirable to reduce the laser damage to a minimum. In our experiments, we used a laser radiant flux of 50 mW and a spot size of 50 μm as in similar reports of other groups, and this resulted in an irradiance of 25 MW / m^2^. In the first description of photochemical induction of thrombosis with rose bengal [34], an irradiance of 6.4 kW / m^2^ was applied for 20 min. This irradiance is 4000 times less than ours what may explain the extent of retinal damage. Therefore, less irradiance should be used in further experiments to reduce damage-induced inflammatory response. 735 W / m^2^ were shown to occlude retinal vessels in rat [35], and this light intensity is only slightly above the physiological range [36,37].

In summary, the effect of laser wounding dominates the response in mice while the relation of the size of the laser spot to the whole retina may be more favorable in larger animals like rat or pig. It is therefore essential to include the appropriate laser wounding control when investigating the gene expression profile of laser-induced vascular occlusion models in small animals. Furthermore, conditions would be necessary where the vein occlusion is achieved without direct laser-induced tissue damage.

## Supporting information

S1 Fig Whole series of sections as shown in Fig 5A. (TIF)

S2 Fig Whole series of sections as shown in Fig 5B. (TIF)
