# Supplementary figures and images for: Gene expression profiling in a mouse model of retinal vein occlusion induced by laser treatment reveals a predominant inflammatory and tissue damage response
Source: PLoS One. 2018 Mar 12;13(3):e0191338. doi: 10.1371/journal.pone.0191338 (PMC5846732; doi:10.1371/journal.pone.0191338)

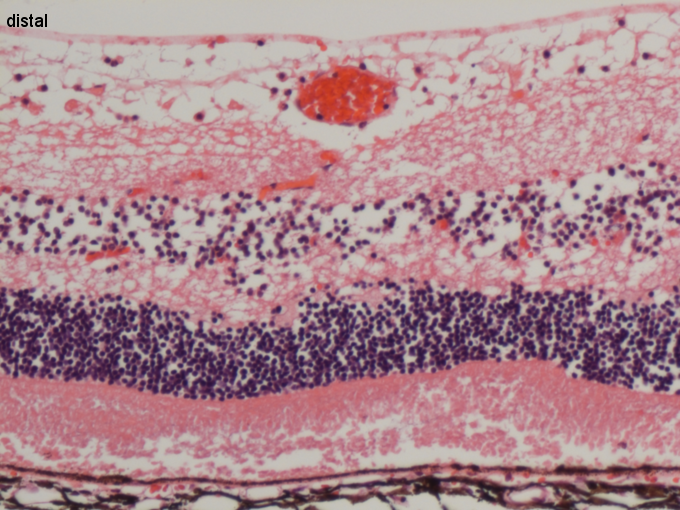

Supplement: S1 Fig — (TIF) [file pone.0191338.s001.tif]
